# Liquid biopsy for children with central nervous system tumours: Clinical integration and technical considerations

**DOI:** 10.3389/fped.2022.957944

**Published:** 2022-11-16

**Authors:** Reda Stankunaite, Lynley V. Marshall, Fernando Carceller, Louis Chesler, Michael Hubank, Sally L. George

**Affiliations:** ^1^ Division of Molecular Pathology, The Institute of Cancer Research, London, United Kingdom; ^2^ Clinical Genomics, Royal Marsden NHS Foundation Trust, London, United Kingdom; ^3^ Evolutionary Genomics and Modelling, Centre for Evolution and Cancer, The Institute of Cancer Research, London, United Kingdom; ^4^ Paediatric Tumour Biology, Division of Clinical Studies, The Institute of Cancer Research, London, United Kingdom; ^5^ Children and Young People's Unit, Royal Marsden NHS Foundation Trust, London, United Kingdom

**Keywords:** cell free DNA (cfDNA), liquid bioposy, paediatric oncology, CNS tumours, molecular diagnostics, personalised medicine, cancer heterogeneity, circulating tumour DNA (ctDNA)

## Abstract

Circulating cell-free DNA (cfDNA) analysis has the potential to revolutionise the care of patients with cancer and is already moving towards standard of care in some adult malignancies. Evidence for the utility of cfDNA analysis in paediatric cancer patients is also accumulating. In this review we discuss the limitations of blood-based assays in patients with brain tumours and describe the evidence supporting cerebrospinal fluid (CSF) cfDNA analysis. We make recommendations for CSF cfDNA processing to aid the standardisation and technical validation of future assays. We discuss the considerations for interpretation of cfDNA analysis and highlight promising future directions. Overall, cfDNA profiling shows great potential as an adjunct to the analysis of biopsy tissue in paediatric cancer patients, with the potential to provide a genetic molecular profile of the tumour when tissue biopsy is not feasible. However, to fully realise the potential of cfDNA analysis for children with brain tumours larger prospective studies incorporating serial CSF sampling are required.

## Introduction

Circulating cell-free DNA (cfDNA) is fragmented genomic DNA that is present in biological fluids. In patients with cancer, a subset of cfDNA is derived from tumour cells and hence is known as circulating tumour DNA (ctDNA). In this review the term ctDNA is used to refer to tumour-derived DNA present in any accessible body fluid, and the term CSF-ctDNA for tumour derived DNA specifically from CSF. When discussing analytical methods, the term cfDNA is more appropriate, as we do not have the tools to separate the ctDNA from cfDNA in the laboratory.

The isolation and molecular profiling of cfDNA has shown great potential for identification of actionable biomarkers in various cancers, especially genomic molecular profiling identifying single nucleotide and copy number changes. Given the inherent difficulties of acquiring tissue biopsies in children with cancer, this less invasive method offers not only the possibility of providing the molecular profile of the tumour when tissue biopsy is not possible but also the potential to assess tumour heterogeneity and monitor disease course and response to treatment through serial sample collection. The evidence for the clinical utility of cfDNA analysis in patients with different types of paediatric tumours has been accumulating, with highest potential in neuroblastoma, osteosarcoma, rhabdomyosarcoma, malignant renal tumours and Ewing’s sarcoma (1–4). However the methodology of the studies exploring the use of liquid biopsies in paediatric cancers has varied, including custom designed droplet digital PCR (ddPCR) assays, custom next generation sequencing (NGS) panels, whole exome sequencing (WES), whole genome sequencing (WGS) and methylation assays. Therefore, the data is currently not sufficient to make specific recommendations for routine implementation into the clinic.

The need for liquid biopsies is very high in patients with central nervous system (CNS) tumours. The ability of neuroimaging to discriminate different brain tumour diagnoses is low, and current practice, as per World Health Organisation (WHO) criteria mandates both histopathological classification and advanced molecular characterisation, which is now considered standard of care (5). For certain CNS tumours, anatomical location precludes diagnostic biopsy, further highlighting the need for non-invasive molecular diagnostics (6). Currently, a combination of magnetic resonance imaging (MRI) (sometimes with functional imaging) and clinical examination is used for diagnosis and to serially assess disease response to therapy, but these have limited sensitivity and specificity, despite international guidelines/consensus (7, 8) and there remains a lack of consistency for defining tumour measurement and response for some tumour types (9, 10). In addition, standard clinical imaging techniques do not facilitate the assessment of molecular changes during therapy or at relapse, which in turn limits treatment options available to these patients.

The potential genetic, epigenetic and protein expression biomarkers that can be evaluated in different paediatric brain tumour types using liquid biopsy tools on circulating tumour cells, miRNA and extracellular vesicles have been reviewed elsewhere (11, 12). In this review we will focus on the most studied genetic markers that can be monitored in cfDNA – single nucleotide (SNV) and copy number variants (CNV). The evidence for CNV and SNV detection in plasma and/or CSF derived ctDNA to aid diagnosis and to identify potentially actionable targets for treatment is rapidly accumulating in multiple tumour types including midline glioma, medulloblastoma, pineoblastoma, atypical rhabdoid/teratoid tumour (ATRT), embryonal tumour with multi-layered rosettes, primary CNS lymphoma and astrocytoma (13–16.) However, in order to fully integrate these assays as clinical diagnostic tools for children with CNS tumours standardisation of sampling, processing and analysis is required alongside larger scale studies defining the sensitivity and specificity and limitations of the different testing methodologies used.

In this review we will discuss the challenges of cfDNA analysis from the blood in patients with CNS tumours and highlight the potential to use CSF as alternative source of cfDNA in these patients. We will discuss the practical considerations of sample collection, processing and analysis and suggest recommendations based on current practice and knowledge, towards the standardisation of these assays in future studies. As larger scale studies will enable the clinical integration of these assays for variant detection, we will also discuss potential future applications, such as the potential to monitor disease course and to evaluate spatial and temporal heterogeneity, with emphasis on CNS tumours.

## Detection of ctDNA in blood lacks sufficient sensitivity in most patients with brain tumours

In most cancers, blood is a good source of circulating tumour DNA and can be used to obtain a molecular profile of the tumour in a minimally invasive way. However, in patients with CNS tumours the ability to detect ctDNA in the blood is much more limited compared to other solid tumours (6, 17). In the paediatric setting, most studies have been limited by small patient numbers and even though they were focused on using highly sensitive methods (mainly ddPCR) that allow detection of variants at very low levels (VAF of 0.01%–0.1%), the ability to detect pathogenic variants in cfDNA from plasma of patients with brain tumours has been low, with 0%–10% of patients having detectable ctDNA alterations in patients with glioma and up to 40% detectability in patients with medulloblastoma (6, 14, 16).

The limited sensitivity of blood-based assays is mainly thought to be due to the blood brain barrier (BBB) that restricts the shedding of ctDNA into the bloodstream. This idea is supported by relatively higher ctDNA levels in high grade glioma (HGG), which is characterised by disrupted BBB and meningioma which grows outside of BBB (18, 19). For example, a recent study showed a sensitivity of 62% (with 90% specificity) for detection of TERT promoter mutations in adult glioma patients as well as potential to track the disease course with serial blood samples in 5 patients (20). Another study of adults with primary brain tumours at various clinical timepoints showed that half of the patients had detectable ctDNA alterations, albeit with average VAF of 0.33% and minimum VAF of 0.05%, highlighting the need for assays with very high sensitivity and specificity (19). However, this was not the case in a large longitudinal study (127 plasma samples from 41 patients) of paediatric non-brainstem HGG where no alterations were detected in any of the patients even though a highly sensitive ddPCR method was used on cfDNA from the blood (21).

Certain CNS tumour types, such as medulloblastoma or ependymoma, can sometimes metastasise outside of the CNS. In these cases, blood based cfDNA profiling may be useful, even before the metastasis occurs, as shown by the presence of circulating tumour cells (CTCs) and ctDNA in several therapy-naïve paediatric medulloblastoma patients (22). However, the largest prospective study of cfDNA from children with CNS tumours, using both ultra-low pass WGS (ULP-WGS) and hybrid capture sequencing with UMIs has further highlighted that detection of ctDNA is limited by low ctDNA fraction and the low numbers of genetic events in these tumours (23).

Overall, while some neurological tumours are potentially more detectable by liquid biopsy than others, detection of ctDNA with clinically informative variants in the blood is inconsistent in patients with CNS tumours.

## Approaches to improve ctDNA detection in blood of brain tumour patients

Several ways to improve the ctDNA detection from the blood of patients with brain tumours have been explored. For example, pre-amplification of cfDNA after extraction has been described as a way to improve the detection of low level ctDNA variants. This led to the detection of ctDNA in 80% of newly-diagnosed patients with glioma from 1 ml of plasma and in 87% of patients from 0.5 ml of cerebrospinal fluid (CSF) (24). In a different study of newly diagnosed patients with diffuse intrinsic pontine glioma, H3K27M mutations were detected in 92% of the patients using the same pre-amplification method (25). The method was further optimised on different ddPCR platforms and tested in multiple laboratories, setting the first steps towards standardisation (26). Mutations were detected in all plasma specimens in this study, but the VAFs were lower in blood than in CSF. However, the low number of samples tested limits the conclusions that can be drawn about the broader applicability of the method. Additionally, it is important to note that while the chances of errors occurring at the specific site during pre-amplification are low, they are not nil. Therefore, while pre-amplification is appropriate for ddPCR based methods, it is likely to introduce higher numbers of false positives if broader NGS based profiling methods (such as NGS panels or WES) are to be used. Assessing individual reads without error correction at pre-defined loci can result in high false-positive rates, highlighting the need to carefully assess sensitivity and specificity of each assay (23). Ultimately sensitivity depends on absolute detection – there must be enough ctDNA molecules for detection.

Another way to increase the sensitivity of detecting variants in cfDNA would be to increase the permeability of the BBB to allow more ctDNA to enter the bloodstream. *In-vivo* and patient studies have both shown that higher ctDNA levels are present after radiotherapy (24, 27). Given that radiotherapy is a mainstay of therapy for most paediatric malignant CNS tumours (with the exception of the youngest patients, for reasons of neurocognitive toxicity sparing) this opens the possibility of planning blood collection for a time when maximal tumour DNA shedding and BBB disruption is predicted. Although this would only provide a snapshot of the molecular profile of a tumour at one specific timepoint during therapy, this may still provide valuable information, particularly if a patient subsequently experiences relapse.

## CSF is a good alternative to blood in liquid biopsies of patients with brain tumours

The low sensitivity and inconsistency of cfDNA profiling from plasma in adult patients with brain tumours led researchers to investigate alternative body fluids as sources of ctDNA. A variety of different profiling methods have been used, with studies showing better detection rates in cfDNA derived from CSF (CSF-ctDNA) than from plasma in a range of CNS tumours, such as HGG and medulloblastomas (23, 28–33.) Studies to date have generally been limited by small patient numbers and restricted availability of fully matched samples, with CSF and plasma often derived from different patients or from different timepoints, hindering statistical analysis. However, a recent study conducted a large prospective analysis of cfDNA obtained from plasma, CSF and urine in 564 specimens from 258 patients with paediatric brain tumours. This study showed best detection potential in CSF but highlighted low detection rates. Ultra low pass WGS detected copy number alterations in 20% of CSF, 1.3% of plasma, and 0% of urine samples, and deep capture panel sequencing detected alterations in 30% of CSF, 2.7% of plasma, and 0% of urine samples) with high-grade tumours showing the best detection for ctDNA in CSF and plasma (23). Nevertheless, an overall trend for higher levels of ctDNA to be present in CSF than in plasma in patients with CNS tumours is emerging (14, 17).

In the paediatric cancer literature, evidence is accumulating that profiling of CSF-cfDNA could be a feasible and efficient tool for the diagnosis and monitoring of paediatric diffuse midline gliomas and medulloblastomas (15, 34). Importantly, in paediatric diffuse midline gliomas CSF-cfDNA profiling highlighted the possibility of detecting pathogenic variants and aiding the inclusion of patients into clinical trials that rely on H3K27M status as a stratification biomarker when biopsy is not feasible (15).

In paediatric medulloblastoma, the largest study so far (123 patients, 476 samples) demonstrated the clinical utility of copy number variant (CNV) detection though low coverage WGS (lcWGS) on CSF-cfDNA and described it as a minimal residual disease (MRD) surrogate marker (35). The detectability of MRD by detection of tumour-associated CNVs in CSF-cfDNA tended to decline with treatment and persistent detection of MRD in CSF-cfDNA correlated with higher risk of relapse (35). Notably, MRD detection using CSF-cfDNA preceded radiographic progression in half of the patients who relapsed (35).

The potential to use CSF-cfDNA to identify the more aggressive subclones driving disease progression has been shown by cases where CSF-cfDNA at baseline was more concordant with the relapsed tumour than with the corresponding primary tumour (35). In addition, the ability of CSF-cfDNA to characterise intra-tumoural heterogeneity was shown in paediatric medulloblastoma patients, where VAF in tissue and CSF-cfDNA had good correlation, indicating that CSF-cfDNA allows detection of small subclones present in the tumour (33) and in paediatric brainstem glioma patients where CSF-cfDNA profiling detected variants not present in the primary tissue sequencing (36).

Despite encouraging signs of utility, it is important to acknowledge that CSF is not as easily obtainable as blood and sample processing is far from standardised. Inconsistent collection and suboptimal processing can lead to poor sample quality. For example, a recent study of paediatric medulloblastoma that failed to detect most of the mutations in CSF-cfDNA that were expected from the tissue sequencing showed that only 15/58 samples had detectable CSF-ctDNA (by fragment size analysis) (37). The ability to compare the different studies in a meaningful way and issue guidelines is further hindered by the variability in time of collection, pre-extraction handling and the collection method used (CSF from lumbar puncture, ventricular shunt, external ventricular drain, and various CSF reservoirs have been tested). However, this is not possible to control for as it is dependent on the specific clinical situation. Therefore, the next part of this review will focus on providing suggestions for optimal sample processing for future studies and highlight the challenges facing the field.

## Practical considerations for liquid biopsy implementation for children with brain tumours

### Considerations for CSF sampling

As discussed above, for the majority of patients with CNS tumours, CSF is a better source of ctDNA than blood. However, obtaining CSF samples is more invasive and demanding than collecting peripheral blood, requiring sedation/anaesthesia, and increasing risk of infection when intraventricular devices are used. The majority of studies to date have relied on sampling at timepoints compatible with existing clinical practice and collected surplus CSF when routine lumbar punctures (LPs) were performed (33, 35). More evidence is required before CSF-cfDNA dedicated LPs can be routinely recommended for follow-up or early diagnosis of relapse or disease response monitoring in patients with CNS tumours. However, as the weight of evidence increases to support the use of CSF-cfDNA, there is scope for evaluation in prospective studies comparing MRI with ctDNA for response assessment and monitoring of relapse. Key questions include how confidently and how much earlier than MRI cfDNA can detect emerging relapse, and whether this indeed makes a difference to patient outcomes if alteration to treatment is considered earlier. This will likely require bespoke clinical trials with permission for research samples at specified timepoints dedicated to CSF-cfDNA profiling.

### Sample processing

**Figure 1:**
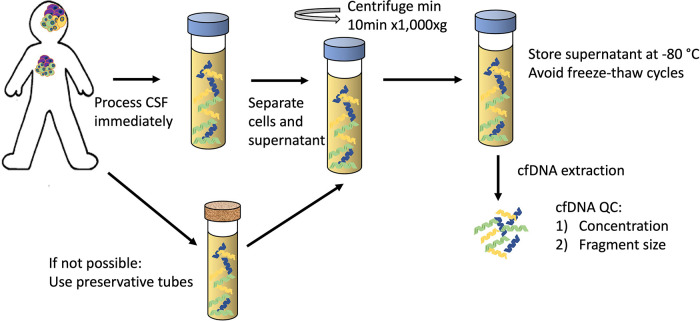
The proposed guidelines for pre-analytical CSF handling for cfDNA analysis.

Success of molecular profiling of cfDNA depends upon maintaining cfDNA integrity and minimising the contamination from genomic DNA from non-cancer cells. Rigorous studies exploring the pre-analytical conditions needed for CSF sample processing for liquid biopsies are lacking, mainly due to the difficulty of acquiring CSF samples from patients. Therefore, the best practices must be inferred from studies in blood and the limited studies we have in CSF. The current best practices of pre-analytical CSF-cfDNA handling, based on the methodologies in the most recent literature (33, 36, 38–40), are shown in Figure 1:

Sample needs to be processed immediately:

• Separate supernatant from the cell pellet (min 10 min x 1,000xg)
• Store at −80°C prior to processing; multiple freeze-thaw cycles should be avoided
• Extraction methods have not been systematically evaluated, most commonly used – Qiagen® “QIAamp Circulating Nucleic Acid Kit”
• Quality Control to assess DNA concentration (Qubit® assay or qPCR-based measurement) and fragment size (by automated electrophoresis systems) to confirm the presence of cfDNA

If immediate processing is not possible, collection tubes with preservative should be used.

Even though CSF has low cellular content, the separation of CSF into the supernatant and the cell pellet is necessary. The cfDNA in the supernatant of CSF has been shown to have higher VAF of cancer-associated variants when compared to the pellet from the same sample, indicating that supernatant often contains sufficient DNA and provides more reliable results than the cellular fraction (40, 41). It is partly explained by the presence of contaminating cells from CSF collection procedure and/or infiltrating lymphocytes, diluting the tumour variant signal in the pellet (41). However, a high proportion of the patients in these studies harboured solid tumours with CNS metastases, and a more extensive confirmation in localised CNS primary tumours would be helpful. The preliminary data points to higher numbers of SNVs and structural rearrangements detected in CSF supernatant in primary CNS tumours (41), therefore, it is routine to spin down the CSF to remove contamination from non-cancer cells, but the exact protocol differs between laboratories - speeds ranging from 500xg for 5 min (26) to 3,000xg for 5 min (33) to 1,900 g for 10 min followed by a further 16,000 g for 10 min (36) have been used. Huang and colleagues (38) have investigated this more systematically (albeit only in one patient) and showed that after 1,000xg × 0 min centrifugation, extracted DNA fragments were exclusively around 150 bp, consistent with cfDNA and at lower speed and shorter time larger fragments were also observed. A systematic study evaluating different CSF centrifugation protocols and their effect on the cfDNA quality and detection of cancer specific alterations is needed, but until then, the standard cell separation protocol of ≥10 min centrifugation at 1,000xg is recommended for future studies.

To minimise the contamination from lysing non-cancer cells when samples cannot be processed immediately, specialist cell-stabilising preservation tubes have been used for blood collection for liquid biopsies. There is a range of tubes on the market, but none are specifically designed for CSF. The only study to date comparing different conservation tubes for CSF samples suffered from the limitations of sample availability showed that Norgen® tubes with phosphate-buffered saline to top up low volume samples had the highest cfDNA yields of the different tubes tested (39).

## Considerations for SNV detection, interpretation in cfDNA and clinical diagnostic application

Whatever the source of cfDNA – blood, CSF or urine – the greater the depth of error-corrected sequencing, the more information about frequency of variants will be retained, up to the point where the number of DNA molecules present in the sample becomes a limiting factor. cfDNA tumour fraction estimates could serve as a guide to the interpretation of plasma cfDNA results, especially negative results, and inform clinical decision making (42). The ability to identify subclones in cfDNA depends on sample purity, ploidy and sequencing depth and to move the field forward, highly sensitive but broad methods, such as panel sequencing or cancer-type specific ddPCR multi-target assays would be needed. Only in high purity samples, WES and WGS should be considered, as achieving sufficient depth in low purity cfDNA samples would become economically unjustifiable.

Depending on the clinical situation, it is likely that cfDNA analysis will be performed without a time-matched tissue sample. It is well-documented in adult cancers that white blood cells also acquire mutations that could mistakenly be profiled as coming from the tumour tissue in blood derived cfDNA analysis (43). The accumulation of these mutations (clonal haematopoiesis of indeterminate potential, or CHIP) is age related (44, 45) and therefore is not normally a significant concern in paediatric cancer patients (46). Nevertheless, accumulation of mutations driving clonal haematopoiesis during cancer therapy have been reported, including paediatric cancer patients (47–50.) Clonal haematopoiesis mutations associated with previous exposure to cancer therapy were most often observed in the DNA damage response and repair genes (TP53, PPM1D and CHEK2) (48, 49), which are also often mutated in paediatric tumours (4, 51). Therefore, caution is needed when interpreting low level variants in cfDNA without matched tissue analysis. This can be done by either analysing the buffy coat in parallel to cfDNA to exclude CHIP derived variants or more rigorous filters on cfDNA at genomic locations commonly associated with CHIP to reduce the risk of interpreting CHIP variants as real tumour-related variants in cfDNA analyses (52, 53). Machine learning algorithms have great potential to help solve this issue in a similar way that that has been done to increase signal enrichment in cfDNA (54).

These complicating factors must all be considered when translating a cfDNA assay to the clinical diagnostic setting. Accreditation and regulatory organisations like the FDA in the US, the MHRA in the UK and the EMA in Europe will require standardisation of sampling and robust clinical validation which is likely to limit the number of laboratories offering cfDNA sequencing as a clinical assay. Commercial providers are already offering CE-marked and CLIA-approved tests for extracranial ctDNA blood testing. These assays are beginning to make the transition from clinical trials into standard of care testing, either through send-away testing or through large regional accredited laboratories like the Genomic Laboratory Hubs in England. To date, however, none offer a CSF-cfDNA focussed method to detect variants, and importantly, to report the absence of, neurological tumours. The commercial market for paediatric tumour testing is also limited, so focussed tests are likely to continue to depend upon translation of academic research assays in this specific field of diagnostics.

## Potential future applications of cfDNA SNV and CNV profiling

### Liquid biopsy methods allow detection of heterogeneity in cfDNA

**Figure 2:**
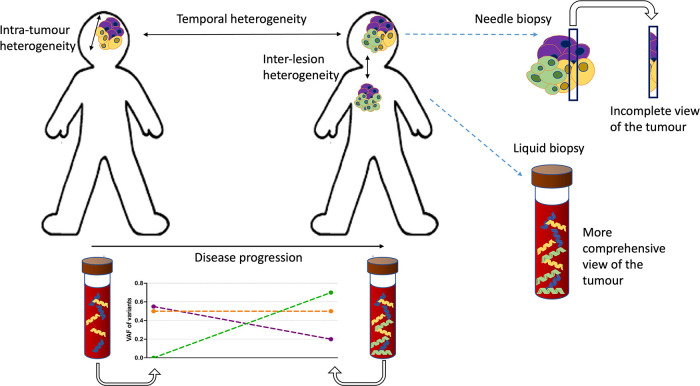
Types of heterogeneity and the advantages of cfDNA profiling. There are two types of tumour heterogeneity: spatial and temporal. Spatial heterogeneity is characterised by the presence of distinct variants in different parts of the same lesion (*intra-tumoural* variation) or between different lesions and metastatic sites in the patient *(inter-lesion heterogeneity)*. Temporal heterogeneity is described as the variation in genetic profile over the time course of the disease. Both types of heterogeneity could potentially be evaluated and monitored using liquid biopsy tools. The temporal heterogeneity could be tracked using serial sampling. The problem of subsampling by tissue biopsies in heterogenous cancers could also be alleviated by liquid biopsy methods that represent the genetic diversity of the tumour better. In this schematic the molecularly distinct (sub)clones of the tumour are indicated in different colours.

A major potential application for liquid biopsy is the ability to detect tumour heterogeneity (Figure 2). Given that solid tumours are spatially heterogeneous, needle-biopsy sampling as a primary means of diagnosis can be problematic (55–57). ctDNA likely originates from a wider sample of the tumour mass and could represent a better surrogate indicator of any inherent heterogeneity, but the utility of this requires further investigation.

Studies in adult malignancies, comparing multi-region and/or multi-lesion tissue sequencing with time matched cfDNA indicate that cfDNA is representative of the diversity of intra-tumoural and intra-lesional heterogeneity (58, 59). However, highly sensitive methods are needed to detect low level sub-clonal variants. Increased shedding of ctDNA from more aggressive, resistant clones into the blood has been reported in adult patients with breast, gastro-intestinal (GI), and lung cancer (60–62). Multi-regional tissue sequencing compared to cfDNA in adult non-small cell lung cancer (NSCLC) (60) and hepatocellular carcinoma (63–65) showed that ctDNA reflects the truncal-branching hierarchy determined by tissue sequencing but it does so incompletely. In the paediatric setting there is very little information to date about the potential of cfDNA to assess the spatial heterogeneity of tumours. In neuroblastoma, where intra-tumoural heterogeneity and ctDNA levels are high, cfDNA profiling might reasonably be expected to represent spatial heterogeneity but the potential that aggressive clones might be overrepresented in higher fractions has been highlighted (4, 66–68). The only study so far looking at multi-region sequencing of high-risk neuroblastoma and comparing it with cfDNA from the blood revealed high intra-tumoural heterogeneity and multiple differences between variants detected in the tissue and cfDNA (69). Deep NGS panel sequencing also highlighted the ability to detect potentially actionable variants in cfDNA that were missed in conventional tissue biopsy profiling (70, 71). Overall, while cfDNA sequencing presents a more complete picture of tumour heterogeneity, there are still uncertainties remaining that limit its application as a diagnostic approach in the clinic.

### Serial cfDNA samples can describe temporal heterogeneity and allow disease monitoring in paediatric cancer patients

Liquid biopsies also have potential for use as a non-invasive means of assessing treatment response and for surveillance for disease relapse. Dynamics of mutation variant allele frequency and/or genome wide copy number profiles in cfDNA are reported to mirror disease progress in diffuse midline glioma and medulloblastoma (13–16). This is also of high relevance in children where tumours are likely to acquire changes between initial diagnosis and relapse (69, 72–76) and serial profiling of cfDNA could identify the emergence of clinically relevant resistance alterations, as reported in adult cancers (60, 77). The benefits of this approach have been shown in several cases in our cohort of paediatric patients with solid tumours, where serial sampling identifies the emergence of potentially targetable alterations at first or subsequent relapse that could have changed the treatment plan (71). For example, in paediatric low-grade gliomas (pLGG), acquisition of CDKN2A deletion at relapse may define a higher risk subgroup and may potentially be targetable (78). To put this into practice, a broad molecular profiling method, such as a targeted panel covering the most common alterations or WES should be used to detect the emergence of new variants. This approach may be of particular importance in CNS tumours, where repeated tissue biopsies are particularly invasive and challenging.

## Future directions and alternative methodologies

Even when pre-analytical challenges are addressed, biological limitations remain - low ctDNA amounts in the blood or CSF and relatively low volumes of CSF collected from paediatric patients limits sensitivity and specificity. This review focuses on the most established cfDNA readouts – SNV and CNV profiling. However, an inherent limitation of the sequencing approaches discussed above is low mutational burden in some paediatric CNS tumours which limits the number of alterations to assess and hence the sensitivity of the assays. Other forms of molecular analysis such as epigenetic, metabolomic and transcriptomic profiling have also been performed in cfDNA from patients with brain tumours (11, 12). Epigenetic profiling is the most advanced of these techniques at the moment and includes methylation, fragmentation, and nucleosome occupation analyses (79). Methylation profiling in particular, is now being routinely used as a diagnostic tool in tissue biopsy samples, and the evidence of its utility on cfDNA is accumulating (80–83). In paediatric sarcoma patients, ctDNA detection and classification based on cancer-specific chromatin signatures and independent of genomic alterations has been successful (83). This can also potentially be applied to CSF-cfDNA and has already been demonstrated in paediatric medulloblastoma where epigenetic signatures were similar in tissue and CSF- cfDNA and retained subtype specificity in patients with good quality samples (81). Dynamic changes in methylation of signature clusters were reported with reduction of methylation levels of cfDNA in patients responding to treatment (81). A proof-of-concept study using reduced representation bisulphite sequencing on cfDNA from plasma and CSF successfully classified 81% of samples from a range of paediatric tumours using less than 10 ng of DNA (82). The study included only 4 CSF samples from patients with CNS tumours but was able to distinguish medulloblastoma from ATRT. If validated in larger cohorts of patients with CNS tumours, these technologies may be of particular relevance in certain paediatric brain tumours such as medulloblastoma and ependymoma which have a low mutational burden.

An attractive alternative to standard sequence-by-synthesis techniques and ddPCR methods for cfDNA profiling is nanopore sequencing. While normally applied as long read technology, Nanopore sequencing can be applied to the short cfDNA fragments rapidly and with lower input, as it depends on the concentration of oligonucleotide ends (84, 85). A proof-of-concept study in pHGG showed 85% sensitivity and 100% specificity in CSF samples and demonstrated correlation of VAF detected in cfDNA and radiological response in a clinical trial (85).

Additionally, cfRNA analysis has recently been applied for cancer detection and profiling of cancer patients (86) and may be expanded to paediatric cancer cohorts. It would be of particular use for example for the detection of certain fusions that are highly characteristic of CNS tumours, such as ZFTA-fused ependymomas or BRAF-fused pilocytic astrocytomas. These constitute the most common type of supratentorial ependymoma and of paediatric low-grade glioma, respectively. Metabolome analyses using liquid biopsies in these patients also has potential for disease monitoring in the future (87, 88), but more evidence is needed.

## Discussion and conclusions

cfDNA analysis can contribute to molecular profiling at cancer diagnosis and relapse. It can aid diagnostic sub-classification and the assignment of risk, prognosis and allocation of targeted therapy in paediatric cancer patients. Due to the anatomic location of the tumours, blood based cfDNA profiling is challenging in patients with CNS tumours and CSF-cfDNA is emerging as a biomarker worthy of further evaluation for molecular profiling, especially in specific tumour types such as medulloblastomas or paediatric high-grade gliomas, in patients with unresectable tumours (such as diffuse midline gliomas), or where repeated tissue biopsies are required.

However, to realise this potential for clinical benefit in children with CNS tumours, CSF-cfDNA tests must be robustly evaluated. Firstly, standardisation of sample processing is needed. Subsequently for each methodology, robust QC measures, normal ranges and diagnostic cut-offs must be defined. This will require larger scale studies comparing the different methodologies for different indications. Standardisation of methodologies across international consortia will be crucial with large-scale implementation and harmonisation best placed within the context of international disease-specific clinical trials. Initiatives like this are already underway, such as the SIOP High Risk Medulloblastoma (89) and SIOP II Ependymoma (NCT02265770) trials.

As the weight of evidence for the technical utility of liquid biopsy assays increases, we must next address where the greatest potential is for added benefit in patients. It is clear for example, that the use of CSF-cfDNA profiling to aid inclusion into clinical trials when biopsy is not feasible is beneficial in the proportion of patients where cfDNA profiling is informative. In terms of disease monitoring, we still need more information to understand how much earlier and with what confidence we can detect emerging relapse in cfDNA when compared to MRI. One advantage of earlier diagnosis of relapse, before a child presents with clinical symptoms is the increased likelihood for eligibility for clinical trials. However, one could also argue that if no alternative therapies are available, earlier diagnosis of relapse may increase harm rather than adding benefit.

It will be challenging to change treatment based on a non-imaging finding, which is not accommodated in current clinical trial designs. At present, inclusion criteria and response assessment endpoints are imaging based, and LPs (for CSF-cfDNA collection) are generally not mandated on clinical trials. However, as clinical trials (such as SIOP Ependymoma II trial (NCT02265770)) are starting to collect CSF at the time of the initial biopsy and after surgery this offers the potential for further validation of these assays, and the opportunity to drive a paradigm change in clinical trial design.

In conclusion, cfDNA analyses, in particular CSF-cfDNA profiling, show great potential for diagnostic and monitoring application, but comprehensive evaluation of these methodologies in ongoing clinical trials is urgently required to maximise the potential for clinical utility of cfDNA assays for children with CNS tumours.
